# 气管和肺干细胞定位及其分子调控机制

**DOI:** 10.3779/j.issn.1009-3419.2010.06.015

**Published:** 2010-06-20

**Authors:** 鑫 金, 穆穆 石, 欣 李, 心善 贾

**Affiliations:** 1 110001 沈阳，中国医科大学附属第一医院超声科 Department of Ultrasound, the First Affiliated Hospital of China Medical University; 2 中国医科大学附属第一医院病理科，中国医科大学基础医学院病理学教研室 Department of Pathology, the First Affiliated Hospital of China Medical University, Department of Pathology, College of Basic Medical Sciences, China Medical University, Shenyang 110001, China

## 1 气管干细胞的概念及特性

干细胞均有三个特性：干细胞长时期具有分裂和自我更新的能力；干细胞没有特化功能，即无法特化任何组织特异性结构而能够行使特定功能；能够产生特化细胞，这个过程被称为分化。此外干细胞在组织中为数甚少，而且绝大多数在静态G_0_期。有关气管干细胞有两种观点：

### 1.1 基底细胞干细胞论

基底细胞表达P63及角蛋白5和14（Keratin5/14, Krt5/14）^[1-5]^。成熟气管组织中的细胞更新率通常很低^[6]^，但除纤毛细胞外，气管上皮损伤能够引起其他细胞迅速增殖^[7]^，组织修复的速度很快。Hong等^[8]^应用谱系追踪法对经萘损伤表达Krt14-CreER细胞的研究表明，某些基底细胞能够进行自我更新，并能生成纤毛细胞和分泌细胞。其次，二氧化硫（SO_2_）吸入法损伤气管上皮后，部分基底细胞长期保持脱氧尿嘧啶（BrdU）标记^[9]^。再次，高度表达Krt5-绿色荧光蛋白（green fluorescent protein, GFP）的基底细胞具备更大的增殖及体外克隆的能力^[3]^。另外，移植受体中，分离的小鼠气管基底细胞可以使受损裸露的气管上皮细胞恢复完整^[10]^。因此认为，基底细胞就是干细胞。

### 1.2 基底细胞祖细胞论

但是也有证据不支持基底细胞就是干细胞的观点。Evansi等^[10]^认为，终末细支气管没有基底细胞，而可由Clara细胞修复；胎儿期发生过程中，气管上皮中基底细胞发生于纤毛和粘液细胞之后，气管柱状上皮初期形成时基底细胞不是必需的；基底细胞与柱状细胞的同增减数量比例不恒定；柱状上皮损伤后，主要反应为粘液细胞而不是基底细胞。此外Avri Delplanqur^[11]^发现，水道蛋白3（AQP3）可表达于基底细胞，通过流式细胞仪，把AQP3（+）的基底细胞与AQP3（-）的纤毛细胞及粘液细胞分离，此后再分别移植，结果AQP3（+）及AQP3（-）细胞群均能再建假复层纤毛柱状上皮。由此证明基底细胞与粘液细胞都能进行克隆分化，因而基底细胞不具备特异性。Daniely等^[2]^在实验中敲除了基底细胞标记物P63，在P63阴性的胚胎气管中，基底细胞消失，但纤毛细胞为主的细胞依然存在，由此证明纤毛细胞为主的柱状上皮细胞不必须由基底细胞转化而来。基底细胞约占气管假复层纤毛柱状上皮的30%，与干细胞所应占据的比例不符；基底细胞表达角蛋白K14、K5，说明其进行了一定程度的分化；宋楠等^[12]^证明稳态下基底细胞不表达提示未分化的*Oct3*/*4.Sox2.Nanog*基因，提示其已有分化；由于非基底细胞群能够再建假复层纤毛柱状上皮，因此认为基底细胞为祖细胞，而不是干细胞。同理，纤毛细胞出现纤毛，为终末分化细胞，而粘液细胞分泌粘液，Clara细胞分泌特殊颗粒，Ⅱ型肺泡上皮细胞含有板层小体，上述细胞都有所分化且具备特异功能，且数量很多，因此认为它们均为祖细胞，而不是干细胞。那么，气管干细胞存在于哪里？如何证明其存在呢？

## 2 气管干细胞的证明方法

### 2.1 慢性标记细胞（LRC）

理论被质疑Borthwick^[9]^通过连续在雄鼠气管内滴注聚多卡醇（polidocanol）或SO_2_吸入法使气管上皮受损，同期隔日腹膜内注射BrdU，95天后在小鼠气管腺导管内发现了慢性标记细胞。排除3天、95天的标记指数（LI）分别为65%、30%。其中，60%慢性标记细胞集中在上段气管腺导管；其余集中在下段气管软骨-软骨粘膜接合处；多数为基底细胞而少数为纤毛细胞，由此提出慢性标记细胞就是干细胞。

尽管上述观点曾被许多杂志引用，但由于不能确定小鼠气管上皮细胞的寿命，故观察95天后的细胞终究为经过几次分裂后的子细胞尚属未知。再者，干细胞所占细胞总数的比例不足10%，那么其中30%的慢性标记细胞中必定混有短暂增殖祖细胞。

2007年，Kiel等^[13]^在Nature杂志发表论文，否定了通过慢性标记细胞判定干细胞的可靠性，经特征标记高度提纯造血干细胞对其进行评估。实验利用环磷酰胺以及粒细胞集落刺激因子处理新生小鼠，给予正常成熟小鼠BrdU 4天-10天后，停用70天。仅少于6%的造血干细胞保留BrdU，而保留BrdU的骨髓细胞中，造血干细胞不足0.5%。该实验结果表明：BrdU作为造血干细胞的标记，其特异性和敏感性均很低，能否将其用于气管干细胞尚须进一步证明。

### 2.2 在G_0_期细胞中寻找干细胞

99%的干细胞处于G_0_期，为什么不在G_0_期中找寻？5-氟尿嘧啶（5-FU）为抗嘧啶类代谢药，属于代谢周期性特异的药物，可使增殖期的气管粘膜细胞变性、坏死、脱落，而对G_0_期细胞并不敏感。2003年，贾心善研究组^[14]^使用5-FU诱发制成离体大鼠气管环严重损伤的模型，经5-FU作用12 h后的气管环上皮几乎全部脱落，残留间隔分布的裸核样细胞呈钉状，位于基底膜上，PCNA染色细胞核均为阴性，证明该细胞为G_0_期细胞；去除5-FU 3 h-6 h后，上述细胞变为扁平细胞；9 h-12 h后，扁平细胞逐渐转变为立方上皮；24 h后，可见到纤毛样结构、粘液分泌^[15]^；48 h后气管被假复层柱状上皮细胞所覆盖。在5-FU的打击下，进入细胞周期的气管粘膜细胞变性、坏死、脱落，初始的基底膜是完整的（经嗜银染色证明），那么在上皮修复过程中，上皮细胞只能来源于残留的抗5-FU的G_0_期细胞。换句话说，干细胞就在其中。笔者认为，G_0_期细胞分为两种。其中已分化细胞将永远退出细胞周期而死亡，而其他再次进入细胞周期的细胞则为干细胞，后者随后转变为扁平细胞、立方细胞，并分化出基底细胞、粘液细胞、纤毛细胞。实验结果^[16-18]^提示，气管干细胞存在于气管抗5-FU G_0_期细胞中。此后又证明上述细胞Hoechst33342荧光染色阴性，ABCG2阳性。在5-Fu诱发人支气管损伤修复的过程中，周莹等^[19]^证明气管干细胞存在于对5-Fu有抗性的气管G_0_期细胞中。同时，采用流式细胞仪方法可富集罗丹明（与Hoechst33342相似）阴性细胞。王琳琳等^[20]^观察了大鼠气管损伤修复过程中ABCG2的动态表达，证明在正常气管上皮中ABCG2为阴性。经5-Fu打击后，ABCG2出现，且在培养6 h-12 h达到高峰，此后随着干细胞的分化而下降。廖爱军等^[21]^经大鼠体内气管损伤修复过程中，气管干细胞定位所得的结果与之相同。

## 3 细支气管及肺干细胞的几种观点

远端支气管不存在基底细胞，Clara细胞来源于经萘损伤后少量存活的耐受或“变异”Clara细胞（Clarav）的增殖与自我更新。在支气管/细支气管交界处存在着紧邻神经上皮小体（NEBs）^[22]^且保留标记的Clara细胞亚群。后者与细支气管/肺交界处均可表达sftpc和scgb1a1^[23]^，能够在萘损伤后进行增殖修复。由于变异Clara细胞不表达Cyp2f2酶（一种可将萘转化为毒性产物的P450细胞色素族），故可耐受萘损伤。这种表达缺陷反映出其相对于多数Clara细胞而言，为分化较低的细胞。变异Clara细胞与更加“经典”的未分化干细胞相比较，仅仅是其所表达的分化细胞标记物不同[降钙素基因相关肽、Clarav细胞分泌的scgb1a1，以及细支气管肺泡干细胞（BASCs）分泌的sftpc和scgb1a1]。

Giangreco等^[24]^利用萘处理小鼠终末细支气管6 h剥离Clara细胞后，经皮下微泵给予[3H]7天。实验者将细胞核内含5个以上银粒的细胞定义为“被标记”细胞，含15个以上银粒的细胞定义为LRCs。45天后，LRCs占全部标记细胞的12%。该实验将终末细支气管干细胞定位于支气管腺泡导管连接处（BADJ）^[23]^。以往的研究中，BASCs的分离及分型多遵照其Sca-1^+^CD45^-^CD31^-^CD34^+^的免疫表型^[25]^。而近期的研究将流式细胞分析与小鼠模型相结合，Hayashi等^[26]^认为上述标记物并不能真正从Clara细胞群中分离出干细胞。新的细胞表型的标记物需要通过细胞显微切割和微阵列分析等多项技术进行证实。经萘损伤后的纤毛细胞能否跨组织类型分化、变异Clara细胞与BASCs是否为稳态或受损组织中唯一具备干细胞潜能的细胞类型，尚无定论。有些模型能以其各自方式摧毁纤毛细胞或Ⅰ型肺泡细胞。摧毁方式包括吸入二氧化氮和臭氧（选择性杀伤纤毛细胞）等氧化剂或服用博莱霉素（杀灭Ⅰ型细胞）等化疗剂。上述研究结果皆支持纤毛细胞可由Clara细胞再生而来，而Ⅰ型细胞可由Ⅱ型细胞所产生。但这些研究结果还需要通过体内谱系学加以证实^[27-29]^。而是否所有的Ⅱ型细胞都能生成Ⅰ型细胞，或是仅在如BASCs等特定区域才有此能力，目前尚无定论。

## 4 气管及肺干细胞的分子调节

这些研究主要集中在基因操控信号通路、肺发育和肺癌发生过程所涉及的分子机制。通过经典的Wnt信号通路，包括β-catenin^[30-32]^或者通过MAP激酶或Ras通路，PTEN^[33]^、GATA-6^[34]^和Bmi1^[35]^的信号途径。这些研究通过基因敲除或使用目的基因干预方法导致靶基因的表达发生改变。通过对β-Catenin信号通路的激活，K-Ras^[36]^信号途径或GATA-6^[37]^、PTEN、PI3激酶或者是p38αMAP激酶^[38]^的丢失，已经发现导致CCSP/Pro-SPC双免疫阳性细胞的增加和富集，有些学者认为这些就是支气管干细胞。将胚胎干细胞特异基因*Oct3*/*4.Sox2.Nanog*导入纤维母细胞或皮肤细胞后产生干细胞样细胞，表明其具有维持未分化状态的能力^[39-44]^。Song等^[12]^最新研究发现，常态气管粘膜上皮不表达Oct3/4.Nanog.Sox2，而经5-Fu处理后，气管粘膜上皮基底膜仅残留G_0_期细胞，却出现Oct3/4.Nanog.Sox2表达。认为这些阳性表达的细胞是通过核再编程，从而转变为干细胞。细胞空白区域填补后，伴随分化，这些阳性表达细胞逐渐减少；恢复假复层纤毛柱状上皮后，上皮细胞恢复Oct3/4.Nanog.Sox2阴性。初步研究结果表明，在受到严重打击后，残存的G_0_期细胞*Oct3*/*4.Nanog.Sox2*基因启动子发生DNA去甲基化，核再编程，表达*Oct3*/*4.Nanog.Sox2*基因，而其本身转化为干细胞，随后*Oct3*/*4.Nanog.Sox2*启动子甲基化，基因沉默，干细胞开始分化，亦即表观遗传学调控干细胞分化。

## References

[1] Evans MJ, Van Winkle LS, Fanucchi MV (2001). Cellular and molecular characteristics of basal cells in airway epithelium. Exp Lung Res.

[2] Daniely Y, Liao G, Dixon D (2004). Critical role of p63 in the development of a normal esophageal and tracheobronchial epithelium. Am J Physiol Cell Physiol.

[3] Schoch KG, Lori A, Burns KA (2004). Asubset of mouse tracheal epithelial basal cells generates largecolonies *in vitro*. Am J Physiol Lung Cell Mol Physiol.

[4] Nakajima M, Kawanami O, Jin E (1998). Immunohistochemical and ultrastructural studies of basal cells, Clara cells and bronchiolar cuboidal cells in normal human airways. Pathol Int.

[5] Boers JE, Ambergen AW, Thunnissen FB (1998). Number and proliferation of basal and parabasal cells in normal human airway epithelium. Am J Respir Crit Care Med.

[6] Kauffman SL (1980). Cell proliferation in the mammalian lung. Int Rev Exp Pathol.

[7] Rawlins EL, Ostrowski LE, Randell SH (2007). Lung development and repair: contribution of the ciliated lineage. Proc Natl Acad Sci USA.

[8] Hong KU, Reynolds SD, Watkins S (2004). In vivo differentiation potential of tracheal basal cells: Evidence for multipotent and unipotent subpopulations. Am J Physiol Lung Cell Mol Physiol.

[9] Borthwick DW, Shahbazian M, Krantz QT (2001). Evidence for stem-cell niches in the tracheal epithelium. Am J Respir cell Mol Biol.

[10] Evans MJ, Shami SG, Cabral Anderson LJ (1986). Role of nonciliated cells in renewal of the bronchial epithelium of rats exposed to NO_2_. Am J Pathol.

[11] Avril-Delplanque A, Casal I, Castillon N (2005). Aquaporin-3 expression in human fetal airway epithelial progenitor cells. Stem Cells.

[12] Song N, Jia XS, Jia LL (2010). Expression and role of Oct3/4. Nanog and Sox2 in regeneration of rat tracheal epithelium. Cell prolif.

[13] Kiel MJ, He S, Ri na, Ashkenazi R (2007). Haematopoietic stem cells do not asymmetrically segregate chromosomes or retain BrdU. Nature.

[14] Jia XS, Ding Q, Zhou Y (2003). Study on the location of tracheal stem cells. Stem Cell and Cellular Therapy.

[15] Ding Q, Jia XS, Zhou Y (2004). Study of tracheal regeneration after injury induced by 5-fluorouracil in rats. Zhonghua Bing Li Xue Za Zhi.

[16] Jia XS, HASUI K (2003). Observation of rat thacheal stem cells during the injure and regeneration indeced by 5-FU. Proc Japan Society Pathol.

[17] Ding Q, Jia XS, Zhou Y (2004). Study of tracheal regeneration after injury induced by 5-fluorouracil in rats. Zhonghua Bing Li Xue Za Zhi.

[18] Ding Q, Jia XS (2004). Observation of rat tracheal stem cells during the injure and regeneration induced by 5-FU. Acta Anatomica Sinica.

[19] Zhou Y, Jia XS, Ding Q (2003). Study of human bronchia epithelium injury induced by fluorouracil and on location of bronchial stem cells. Chin J Histochem Cytochem.

[20] Wang LL, Jia XS (2006). Expression of ABCG2 transporter during tracheal regeneration in rats. Acta Anatomica Sinica.

[21] Liao AJ, Jia XS, Qu Y (2005). Study of rat tracheal regeneration after injury *in vivo* and on location of bronchial stem cells. Chin J Histochem Cytochem.

[22] Rawlins EL, Okubo T, Xue Y (2009). The role of Scgb1a1^+^Clara cells in the long-term maintenance and repair of lung airway, but not alveolar, epithelium. Cell Stem Cell.

[23] Hong KU, Reynolds SD, Giangreco A (2004). Clara cell secretory protein-expressing cells of the airway neuroepithelial body microenvironment include a label-retaining subset and are critical for epithelial renewal after progenitor cell depletion. Am J Respir Cell Mol Biol.

[24] Giangreco A, Reynolds SD, Stripp BR (2002). Terminal bronchioles harbor a unique airway stem cell population that localizes to the bronchoalveolar duct junction. Am J Pathol.

[25] Kim CF, Jackson EL, Woolfenden AE (2005). Identification of bronchioalveolar stem cells in normal lung and lung cancer. Cell.

[26] Hayashi T, Ishii A, Nakai S (2004). Ultrastructure of goblet-cell metaplasia from Clara cell in the allergic asthmatic airway inflammation in a mouse model of asthma *in vivo*. Virchows Arch.

[27] Aso Y, Yoneda K, Kikkawa Y (1976). Morphologic and biochemical study of pulmonary changes induced by bleomycin in mice. Lab Invest.

[28] Barth PJ, Muller B (1999). Effects of nitrogen dioxide exposure on Clara cell proliferation and morphology. Pathol Res Pract.

[29] Evans MJ, Cabral LJ, Stephens RJ (1975). Transformation of alveolar type 2 cells to type 1 cells following exposure to NO_2_. Exp Mol Pathol.

[30] Jia XS, HASUI K (2006). Stem cell proliferation, differentiation process of the trachea Wnt pathways. Proc Japan Society Pathol.

[31] Lv WL, Jia XS (2006). Expression of Wnt-1 in rat tracheal stem cell during the early proliferation and differentiation. Acta Anatomica Sinica.

[32] Reynolds SD, Zemke AC, Giangreco A (2008). Conditional stabilization of betacatenin expands the pool of lung stem cells. Stem Cells.

[33] Yanagi S, Kishimoto H, Kawahara K (2007). Pten controls lung morphogenesis, bronchioalveolar stem cells, and onset of lung adenocarcinomas in mice. J Clin Invest.

[34] Zhang Y, Goss AM, Cohen ED (2008). A Gata6-Wnt pathway required for epithelial stem cell development and airway regeneration. Nat Genet.

[35] Dovey JS, Zacharek SJ, Kim CF (2008). Bmi1 is critical for lung tumorigenesis and bronchioalveolar stem cell expansion. Proc Natl Acad Sci USA.

[36] Jackson EL, Willis N, Mercer K (2001). Analysis of lung tumor initiation and progression using conditional expression of oncogenic K-ras. Genes Dev.

[37] Ma XB, Jia XS, Liu YL (2009). Expression and role of Notch signaling in the regeneration of rat tracheal epithelium. Cell prolif.

[38] Ventura JJ, Tenbaum S, Perdiguero E (2007). p38α MAP kinase is essential in lung stem and progenitor cell proliferation and differentiation. Nat Genet.

[39] Babaie Y, Herwig R, Greber B (2007). Analysis of Oct4-dependent transcriptional networks regulating selfrenewal and pluripotency in human embryonic stem cells. Stem Cells.

[40] Takahashi S, Yamanaka (2007). Induction of pluripotent stem cells from adult human fibroblasts by defined factors. Cell.

[41] Giangreco A, Groot KR, Janes SM (2007). Lung cancer and lung stem cells: strange bedfellows?. Am J Respir Crit Care Med.

[42] Snyder JC, Teisanu RM, Stripp BR (2009). Endogenous lung stem cells and contribution to disease. J Pathol.

[43] Jia XS (2000). Recent molecular pathology of lung cancer research and prospect. Zhonghua Bing Li Xue Za Zhi.

[44] Xiong YL, Jia XS (2010). Progress in lung cancer stem cell research. Chin J Lung Cancer.

